# Workshop report - interdisciplinary metabolomic epidemiology: the pathway to clinical translation

**DOI:** 10.1007/s11306-024-02111-4

**Published:** 2024-05-21

**Authors:** Krista A. Zanetti, Lining Guo, Deeba Husain, Rachel S. Kelly, Jessica Lasky-Su, David Broadhurst, Craig E. Wheelock

**Affiliations:** 1 Office of Nutrition Research, Division of Program Coordination, Planning, and Strategic Initiatives, Office of the Director, National Institutes of Health, Bethesda, MD USA; 2 Cary, NC USA; 3 Mass Eye and Ear, Department of Ophthalmology, Harvard Medical School, Boston, MA USA; 4 Channing Division of Network Medicine, Brigham and Women’s Hospital, Harvard Medical School, Boston, MA USA; 5 School of Science, Edith Cowan University, Joondalup, Perth Western Australia; 6 Unit of Integrative Metabolomics, Institute of Environmental Medicine, Karolinska Institutet, Stockholm, Sweden; 7 Department of Respiratory Medicine and Allergy, Karolinska University Hospital, Stockholm, Sweden

**Keywords:** Metabolomic epidemiology, Interdisciplinary research, Clinical translation

## Abstract

Metabolomic epidemiology studies are complex and require a broad array of domain expertise. Although many metabolite-phenotype associations have been identified; to date, few findings have been translated to the clinic. Bridging this gap requires understanding of both the underlying biology of these associations and their potential clinical implications, necessitating an interdisciplinary team approach. To address this need in metabolomic epidemiology, a workshop was held at Metabolomics 2023 in Niagara Falls, Ontario, Canada that highlighted the domain expertise needed to effectively conduct these studies -- biochemistry, clinical science, epidemiology, and assay development for biomarker validation -- and emphasized the role of interdisciplinary teams to move findings towards clinical translation.

## Introduction

A primary objective of metabolomic epidemiology research is to understand the biochemical ramifications and potential clinical implications of metabolomic investigations of human health and disease (Fuller et al., 2023). The power of large metabolomic epidemiology studies has enabled the identification and validation of multiple robust associations between metabolites and phenotypes (Buergel et al., 2022; Kachroo et al. 2022); however, promising findings often fall short of effective translation due to the lack of domain specific expertise required to bridge the gap between observed biochemical perturbation and clinical application. To better position research teams to design biologically and clinically meaningful studies, it is imperative that there is a dialog between domain experts prior to and throughout the study. To date, the practical implications of interdisciplinary research in the context of metabolomic epidemiology has not been fully explored.

**Fig. 1:**
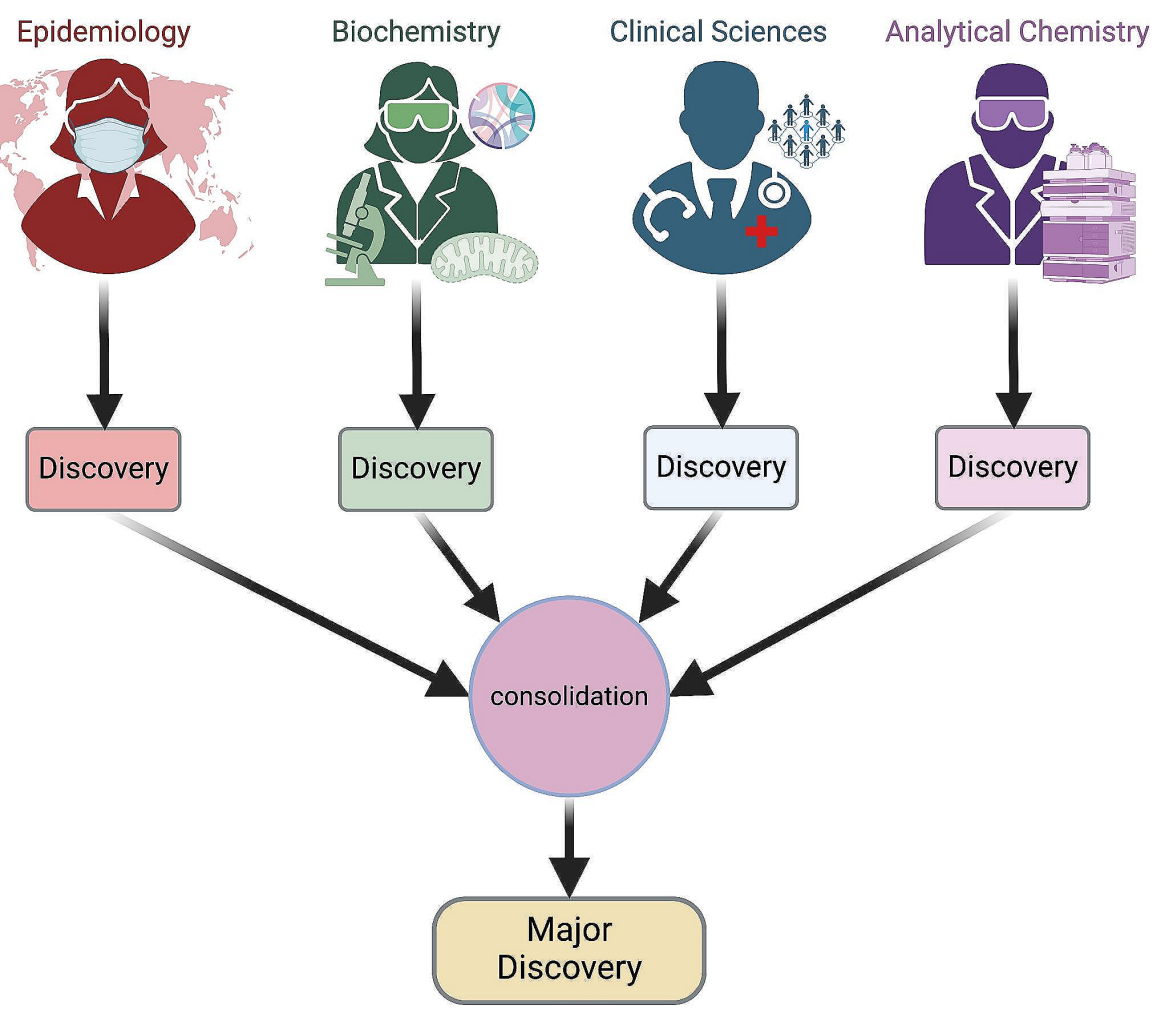
Schema describing the flow of multidisciplinary research from discovery in each discipline to a resulting major discovery. Created with BioRender.com

Frequently, researchers work in multidisciplinary teams when an interdisciplinary approach would be more beneficial. Multidisciplinary teams are a product of different disciplines with shared goals working on a research question independently, often consecutively (Fig. 1). As such, a given research question is examined in isolation from the perspective of each discipline, and although the findings are complementary, essential nuances of knowledge shared across disciplines can often be “lost in translation” (North Carolina State University Research Development Office, 2020).

**Fig. 2:**
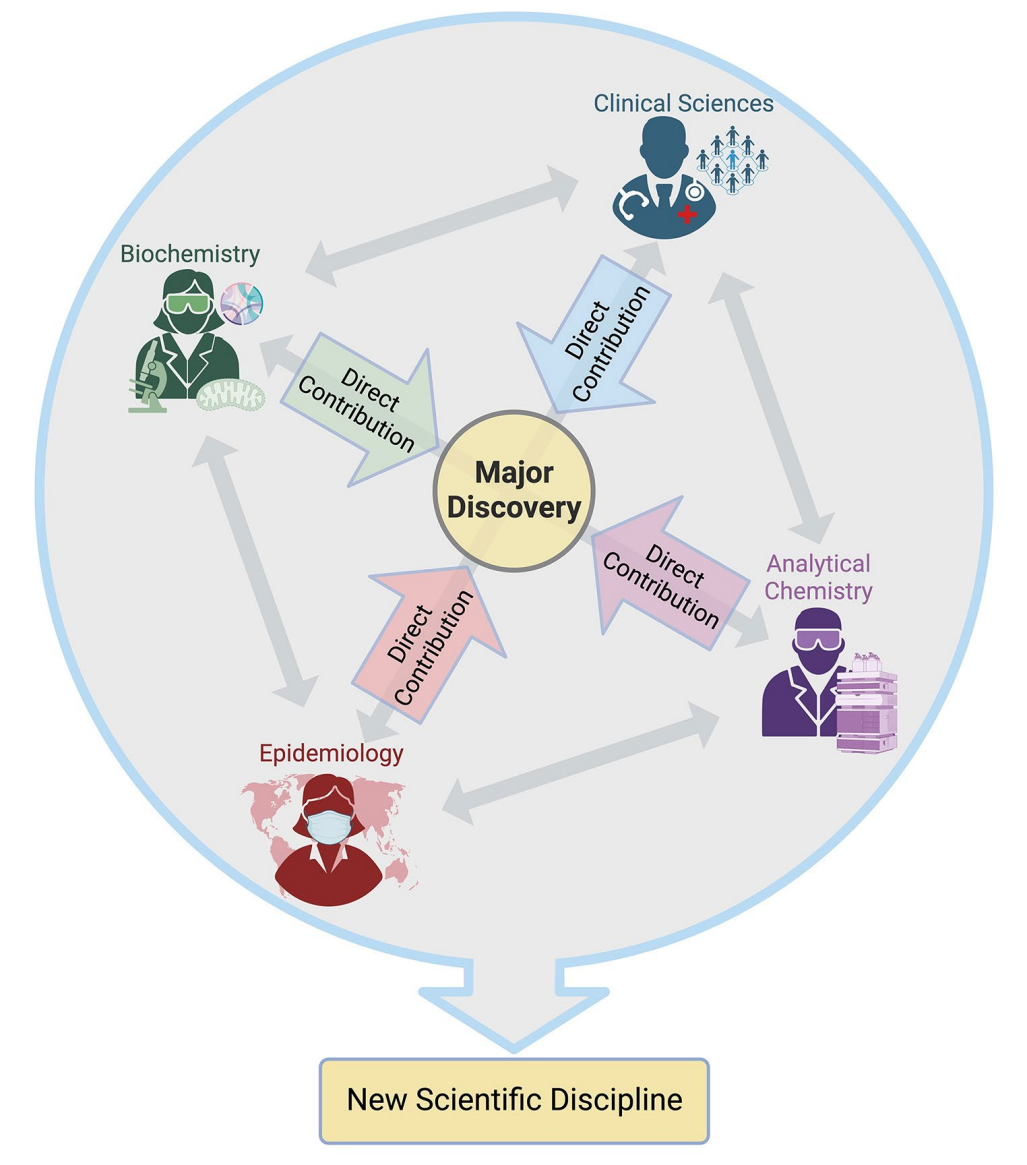
Schema describing the flow of interdisciplinary research from discovery by an integrated team to a resulting major discovery, ultimately resulting in the emergence of a new scientific discipline. Created with BioRender.com

**Fig. 3:**
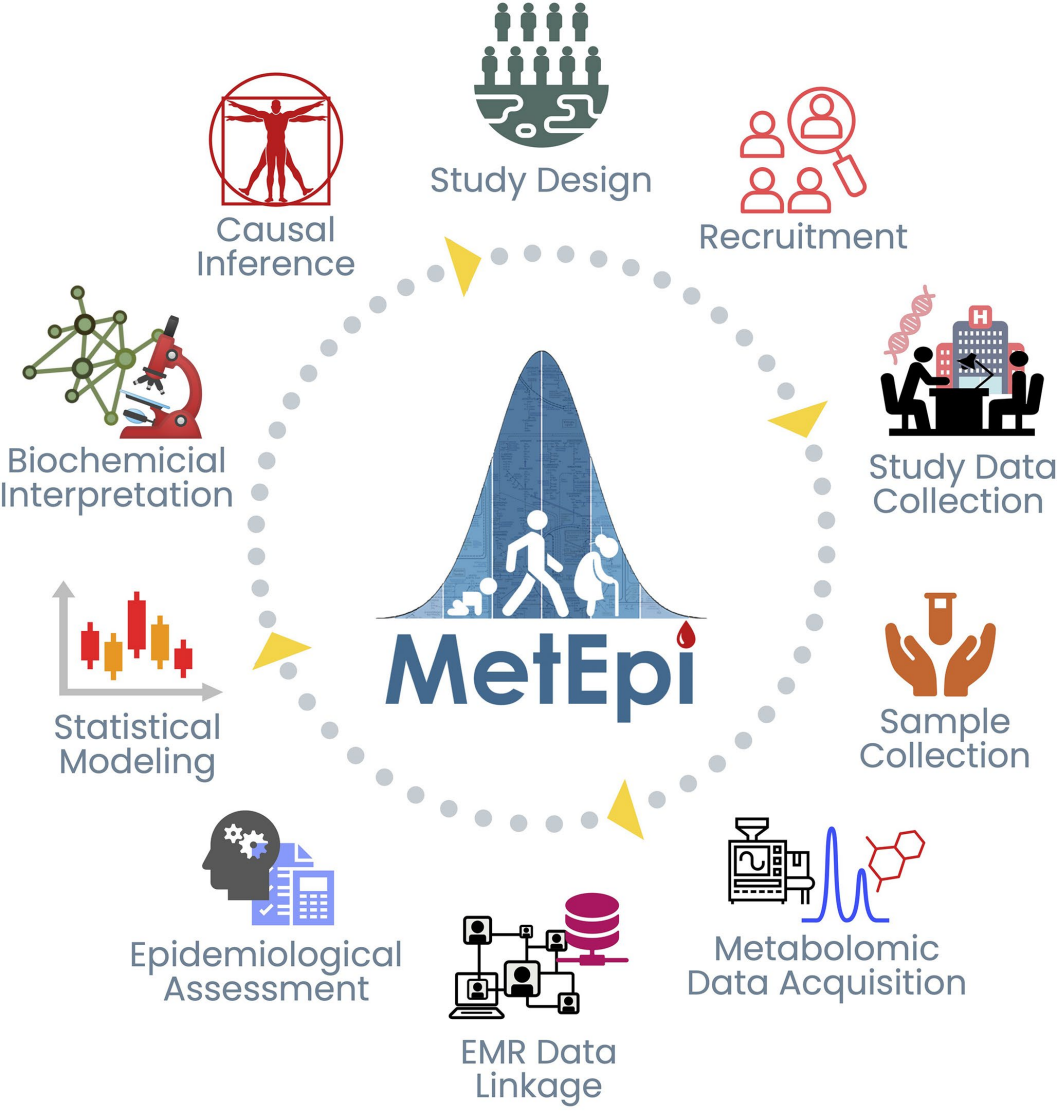
Components and typical workflow for metabolomic epidemiology. Created with BioRender.com

Although multidisciplinary teams are of value, a more effective path to clinical translation is to move towards interdisciplinary teams that interact, work collaboratively, and rely on shared knowledge (Fig. 2). The National Academies defines interdisciplinary research as “*a mode of research by teams or individuals that integrates information, data, techniques, tools, perspectives, concepts, and/or theories from two or more disciplines or bodies of specialized knowledge to advance fundamental understanding or to solve problems whose solutions are beyond the scope of a single discipline or area of research practice *(National Academy of Sciences, National Academy of Engineering, and Institute of Medicine, 2005).” Over time, this framework can lead to a fundamental shift in research practices resulting in the emergence of a new interdisciplinary field. An example of a field that inherently requires interdisciplinary teams is metabolomic epidemiology (Fig. 3) (Lasky-Su et al., 2021).

To date, within the field of metabolomic epidemiology, few findings have been translated to the clinic. Bridging this gap requires a collective and integrated understanding of all aspects of metabolomic epidemiology. As such, the limited capabilities of analytical platforms, the uncertainty of acquired data, and the complex biochemistry of any observed associations must be understood by epidemiologists and clinicians for clinical translation. Conversely, the requirements for population study design, epidemiological inference, and domain-specific clinical installation must be appreciated by chemists, data scientists, and computational biologists. Critically important in this process are the collaborative efforts from a wide array of domain experts to expand overlapping expertise and work as a collective entity. If the research teams collaborate in an interdisciplinary manner, it will inherently increase the probability of clinical translation.

To address the need for interdisciplinary teams in metabolomic epidemiology, a workshop was organized by the Metabolomics Society Metabolomic Epidemiology Task Group and presented at Metabolomics 2023 in Niagara Falls, Ontario, Canada. The workshop aimed to (1) describe the important roles that distinct domain experts play in the process from discovery to clinical translation in metabolomic epidemiology, (2) identify important areas where communication between domain experts is currently lacking, and (3) identify specific steps that can be taken to better establish an interdisciplinary team for metabolomic epidemiology research that will promote the move from initial discovery to clinical translation. Four domain experts provided their perspective on the importance of their distinct roles in metabolomic epidemiology interdisciplinary collaborations to enable translation to the clinic. The presentations highlighted: (1) the role of biochemistry in metabolomic epidemiology, (2) the role of the clinician in metabolomic epidemiology, (3) the role of assay development for biomarker validation in metabolomic epidemiology studies, and (4) the role of interdisciplinary teams to move metabolomic epidemiology studies towards clinical translation.

## Role of biochemistry in metabolomic epidemiology

The complexity of metabolomics data can render it challenging to interpret and place within a biological context. To make sense of this multi-dimensional information, metabolite concentrations must be understood in the context of their biochemical reactions and pathways, being careful not to assume biochemical causation based on observed correlation. Metabolomics can be thought of as the omics science of biochemistry, the study of chemical processes in living organisms. The study of the metabolome offers a global approach to understanding the regulation of metabolic pathways and networks of a biological system. Accordingly, biochemical knowledge is a fundamental component of metabolomic epidemiology and a key requirement of an interdisciplinary approach.

Basic knowledge in biochemistry and physiology can assist in understanding how pathways function and explain why levels of a given metabolite are outside their normal range (i.e., dysregulated). In addition, an understanding of biochemistry is required to predict how to alter chemical inputs in order to achieve a desired change in metabolism (e.g., diet, physical activity). Determining the effects of environmental exposures upon human health – the so-called exposome – also relies upon an awareness of biochemical processes (Zhang et al., 2021). This collective knowledge constitutes the discipline of biochemistry, and metabolomics is ideally poised to translate this information into an actionable strategy to address current health challenges.

When evaluating metabolomics data, biochemical knowledge can be useful in ascertaining the quality and plausibility of the findings. These concerns can be essentialized by a few key domains:

1. Analytical biochemistry - Is the analytical method employed appropriate for the identified metabolite (e.g., positive vs. negative ionization, reversed-phase vs. hydrophilic chromatography, gas vs. liquid chromatography). Is the method employed to extract/detect/quantify the reported metabolite appropriate for the chemical structure? These parameters can enable assessment of whether the analytical method employed can realistically detect the reported compound.
2. Endogenous levels - Is the metabolite produced by the selected organism? Is the metabolite present in the selected matrix? Is the metabolite present at an endogenous level that is detectable by the chosen methodology? Is the metabolite chemically stable? These parameters provide strong guidance on whether a reported metabolite can realistically be present in that sample. For example, while cortisol is a major endogenous steroid hormone in humans, it is not produced in appreciable levels in rats or mice (Gallo-Payet, & Battista, 2014).
3. Isomerism - How many compounds are possible based upon a measured molecular formula (i.e., structural isomers)? Does the stereochemistry of a given chiral center in the detected compound have an *R* or *S* configuration (i.e., stereoisomers)? Is the configuration of a double bond *cis* or *trans* (geometric isomers)? Isomerism is often ignored in metabolomics data due to the inherent chemical complexity combined with a lack of specificity in the analytical methods, potentially resulting in compound misidentification, which can lead to reduced or incorrect interpretation of biochemical findings. However, isomers can provide important information on the synthetic source of the metabolite (e.g., enzymatic vs. autooxidation) and have implications for health. For example, consideration of the *cis* vs. *trans* geometry of double bonds is important for understanding the health effects of dietary fatty acids, with trans fat generally considered to increase cardiovascular risk (Mozaffarian et al., 2006). An infamous example of the importance of stereochemistry is that of thalidomide. This compound is a racemic mixture of two enantiomers (*R* and *S*) that was originally marketed for sleeplessness and morning sickness; however while the *R*-isomer has sedative biological activity, the S-isomer is a teratogen (Smith, 2009). Another example is that of albuterol, which is a chiral short-acting β2 adrenergic receptor agonist used for acute treatment of asthma attacks. *R*-albuterol has bronchodilator and bronchoprotective properties as well as inhibits the activation of eosinophils and mast cells, while the *S*-enantiomer has been reported to intensify bronchoconstrictor responses and induce hypersensitivity of asthmatic airways (Page, & Morley, 1999). Taken together, these biochemical metrics can assist in understanding the likelihood of observing a given metabolite (which may be particularly important in attempts to replicate an existing finding) as well as in interpreting its biochemical and physiological function.

Strictly speaking, in the sense of complete coverage, there is no such thing as “untargeted metabolomics”. Although it is the accepted term for system-wide acquisition of metabolite levels without targeting specific pathways (i.e., hypothesis-free vs. hypothesis-driven metabolomics), all metabolomics datasets are biased by choice of analytical method and constrained by limits of detection, chromatographic separation, and data processing software. Accordingly, the techniques employed for a given project impart an implicit bias in the compounds that can be detected (e.g., lipids vs. small polar organics). This application bias can be particularly relevant for pathway mapping, which is the primary approach to place findings within a biochemical context. The number and proportion of metabolites on a pathway detected will affect the mapping results and misidentifications will skew the findings. For example, detailed analyses have shown that metabolite misidentification results in both gain and loss of significance when conducting pathway enrichment analyses (Wieder et al., 2021).

While biochemical diseases such as phenylketonuria are well-known, in fact the basis for most disease is biochemical in nature due to improper substrate balance (i.e., diet, nutrition) or defective enzyme activity (i.e., pharmacology, genetics) (Barr, 2018). All these influences act to dysregulate normal metabolism. While metabolomics offers a quantitative assessment of metabolism, biochemical knowledge is vital for correct interpretation and health ultimately depends upon the appropriate regulation of biochemistry. Accordingly, lack of biochemical literacy is a major challenge in translating the results of metabolomic epidemiology to the clinic. Dynamic collaborations between biochemists and other investigators (i.e., clinicians, epidemiologists, and statisticians, etc.) are often lacking. There is a need for communication between these different domain experts in order to navigate metabolomic data generation, analysis, and interpretation for effective clinical translation in order to realize the potential of metabolomic epidemiology.

## Role of the clinician in metabolomic epidemiology

Many chronic human diseases are multifactorial and influenced by a combination of genetic, environmental, and lifestyle factors (Oresic et al., 2020), which manifest through biochemical dysregulation (Sobrin, & Seddon, 2014). While a biochemist’s primary unit of interest is the metabolite and its place within the wider metabolic network, the clinician’s primary unit of interest is the patient and it is, therefore, crucial in the determination of whether that metabolite has clinical relevance. The communication between clinicians, epidemiologists, and analytical chemists is currently lacking, which can delay the translation of metabolomic discoveries into clinically relevant biomarkers and treatment targets. In the workshop, it was described how an interdisciplinary team’s role was critical to evaluate the role of metabolomics in age-related macular degeneration (AMD).

AMD is a leading cause of blindness worldwide in people over the age of 50 years (Wong et al., 2014). There are several critical unmet needs of this blinding disease, including the lack of accessible and reliable biomarkers for both diagnosis and prognosis and lack of treatment for the early and intermediate forms of the disease which constitute 90% of AMD (Klein et al., 2014). Metabolomics has been employed in epidemiological studies to help address these gaps (Han et al., 2023).

An epidemiological study to investigate AMD was built from the ground up with the planning phase requiring collaboration between *clinicians with expertise in AMD* and *epidemiologists with expertise in metabolomics*. This led to plans for study design, sample collection, processing and storage, statistical analysis methods, and institutional approvals. Discussions with *analytical chemists* were necessary to determine whether nuclear magnetic resonance or mass spectrometry was the appropriate method of analysis. This collaboration led to a prospective longitudinal study of patients with AMD and controls over age 50 with no retinal disease with a *clinical research team* recruited to perform the study. Information on study subjects was collected, including medical history, diet, and lifestyle, physical exams were completed, and color photos and ocular coherence tomography imaging of participants’ retinas were taken to document retinal structure. Additionally, optional retinal function testing was performed, and morning fasting samples of blood and urine were collected with standardized processing and freezing protocols applied.

After data collection, a *biostatistician* analyzed the pilot study, which showed significant association of AMD with lipids (Lains et al., 2018). In collaboration with *metabolomic epidemiologist*s and *genetic epidemiologists*, a larger validation study was designed using two cohorts to answer the question: are there any metabolomic-genomic associations with AMD that can further our understanding of AMD risk genes? The results of this study showed a significant association of two AMD SNPs with metabolites (Lains et al., 2021), which prompted further investigations to better classify AMD to identify unique phenotypes based in pathophysiology. Retinal structure captured through retinal imaging, and the association between retinal structure and metabolites was examined. This stage of the study required collaboration with *retinal imaging experts*.

The next scientific question asked whether there are metabolic driven endotypes of AMD. In collaboration with an *artificial intelligence expert*, a machine learning method called similarity network fusion was performed, which led to the identification of four metabo-driven endotypes (Personal Communication). These would be the basis for more meaningful, pathobiology-driven classification of AMD and treatment targets for each stage based on specific dysregulated biology, especially for early AMD for which there no treatment at this stage.

This exciting interdisciplinary collaboration and regular communication between the different domain experts has led to novel discoveries. Future studies will examine human eye tissue using targeted metabolomics, which will require new expertise in donor tissue harvesting by *eye banks* and tissue imaging, processing, and interpretation by *ocular pathologists*. There is immense clinical relevance of metabolomics in these studies because it improves understanding of disease pathogenesis, points to potential biomarkers and druggable targets of disease, and can help usher the era of precision medicine in multifactorial diseases.

## Role of the assay development for biomarker validation in metabolomic epidemiology studies

Although metabolomics is a powerful tool for biomarker discovery, it is currently not suitable for direct clinical applications due to its long turnaround time, high cost, and the nature of relative quantitation. Additionally, “untargeted” metabolomics platforms are generally developed to broadly detect as many metabolites as possible in any given sample. As such, the methods may not be optimized for the identification and quantitation of all metabolites. For clinical translation, it is necessary to validate metabolomics findings from epidemiological studies with assays developed to provide absolute quantification of specific metabolites or metabolite classes.

The development of targeted assays involves interdisciplinary efforts, with contributions from biochemists, analytical chemists, and clinicians necessary to optimize an assay. For a given study, it is often the case that multiple potential metabolite biomarkers can be identified through metabolomics analysis. There are several considerations for selecting and prioritizing biomarkers for targeted assay development. The input from a biochemist as to the biological significance and mechanistic insight of biomarkers is crucial. Metabolomic perturbations often impact multiple pathways and produce highly correlated changes in metabolites, which can be utilized by a biochemist to optimize biomarker selection. There are also practical considerations for clinical application of a biomarker. For example, are the biomarkers stable during sample collection? Are chemical standards and isotopically labeled internal standards available for developing high quality assays? What is the cost effectiveness of the targeted assays? One will also need to consider if the targeted assay can meet the requirements of regulated laboratory environments (e.g., compliance to Clinical Laboratory Improvement Amendments/College of American Pathologists guidelines in clinical laboratories or U.S. Food and Drug Administration and International Council for Harmonisation of Technical Requirements for Pharmaceuticals for Human Use guidelines in the pharmaceutical industry (Centers for Medicare & Medicaid Services; European Medicines Agency, 2022; U.S. Department of Health and Human Services Food and Drug Administration Center for Drug Evaluation and Research Center for Veterinary Medicine, 2018).

A recent publication on acute on chronic liver failure (ACLF) provides an interesting case study on the route from metabolomics analysis to proof-of-concept clinical tests (Zhang et al., 2023). ACLF is a clinical syndrome of high short-term mortality in patients with chronic liver disease. Early diagnosis and accurate prognosis can play key roles in ACLF disease management. The authors analyzed over 1,000 plasma samples collected from a muti-center study using untargeted metabolomics. A large majority of the plasma metabolites analyzed in the study were found to be altered by ACLF, highlighting the impact of severe liver disease on the plasma metabolome. The metabolic changes could be explained by several major biological themes such as mitochondrial dysfunction, defective membrane lipid biosynthesis, elevated oxidative stress and inflammation, and altered gut microbiome. The combination of biochemical interpretation of data, machine learning, and availability of chemical and isotopically labeled standards enabled the authors to focus on four metabolites for targeted assay development. After further validation, these assays may have the potential to assist clinicians in improving outcomes for ACLF patients (Badal et al., 2023).

## Interdisciplinary teams to move metabolomic epidemiology study findings toward clinical translation

There are many important considerations for bringing potential metabolite biomarkers identified in epidemiological studies into the clinic. Initial findings from a metabolomic epidemiological study may identify potential metabolite biomarkers via association studies, machine learning, or other analytic approaches that suggest these metabolites might be important for disease prediction, diagnosis, or prognosis. Regardless of their end use, the necessary follow-up step after initial identification is to ensure that the findings are robustly replicated across the spectrum of population characteristics that the biomarker would be applied to, including race/ethnicity, age, sex, BMI, comorbidities, etc. To do this properly, it is essential to have domain experts in epidemiology and statistics as a part of the interdisciplinary team. Early on in this process, communication with analytical chemists is also important as it will provide valuable feedback on the chemical properties that might impact the suitability of each metabolite as a viable biomarker.

While metabolomic epidemiological studies are a powerful way to identify novel biomarkers, it is important to recognize that these are observational studies, and by nature, are not randomized. Therefore, these studies are vulnerable to various types of bias, most notably, confounding. This is particularly true with a case-control design. Observed associations between individuals with and without prevalent diseases may be due to indications of the disease itself. For example, individuals with asthma may use inhaled or oral steroids, so observed differences in metabolite levels may be driven by medication use and not the disease itself (Kachroo et al. 2022). Very often, as in this example, it takes subject matter expertise from the clinician to identify these indications and communication with the epidemiologist to properly account for them. This type of confounding is less likely when a prospective study design is applied; however, considerations of other types of bias persist, making it imperative to assess this by adjusting for various potential confounding variables.

To overcome potential biases, experimental study designs such as randomized controlled trials should be implemented to confirm the observed associations. Such study designs would likely differ from a typical clinical trial but would be designed to address potential confounders and further confirm the viability of the biomarker. Once these factors are verified, candidate biomarkers should then be vetted for their biological relevance to the physiologic state in which they will be implemented. Sometimes the biochemistry of the biomarker is clear because its levels directly relate to the biology and pathophysiology of the disease, (e.g., glucose measures for diabetes). Other times, the biomarker may be less directly involved, yet still related via known biology (e.g., use of serum C-reactive protein to monitor cardiovascular risk (Ridker, 2003). Regardless, the identification of a biomarker is more compelling when there is some mechanistic connection to the disease that can be understood.

There are, however, times where the disease connection may not be immediately clear. If this is a small number of metabolites, then further work may be needed to understand the relationship between the potential clinical biomarker and the biology. There are other cases where the biomarker may be a linear combination of multiple metabolites, which may make it more difficult to connect directly to the underlying biology. However, the biomarker may still have important predictive ability. In such cases, it may not be critical to understand the underlying biology. Following this, careful biomarker development of the potential candidates is necessary as outlined above where the role of the assay development for biomarker validation in these studies is described. In some cases, viable biomarkers, or the metabolites themselves, may already be in use in the clinic which could expedite this process. While examples of this are not common, they are also not unheard of. Examples include using a current clinical biomarker for an alternative purpose, such as measuring lipoproteins for an alternative disease outcome.

## Conclusion

This workshop highlighted the importance of moving from a multidisciplinary approach for metabolomic epidemiology studies to an interdisciplinary approach. This approach enables domain experts to work together by integrating relevant information, data, techniques, and tools, and to share their unique perspectives in real time. Further, it can result in advancements beyond what could be achieved using a multidisciplinary approach, where experts work independently and share data information and data via publications and conference presentations. Collective efforts from an interdisciplinary approach are required to realize the potential of metabolomic epidemiology to understand disease etiology and be effectively translated to the clinic.

## Data Availability

This article reports on a workshop; thus, no new data was generated.

## Declarations

### Software/database availability

This article reports on a workshop; thus, no software or databases were generated.

## References

[CR1] Badal BD, Cox IJ, Bajaj JS (2023). Are we ready to translate metabolomics into clinical practice for ACLF prediction and diagnosis?. Journal of Hepatology.

[CR2] Barr AJ (2018). The biochemical basis of disease. Essays in Biochemistry.

[CR3] Buergel T, Steinfeldt J, Ruyoga G, Pietzner M, Bizzarri D, Vojinovic D, Upmeier Zu Belzen J, Loock L, Kittner P, Christmann L, Hollmann N, Strangalies H, Braunger JM, Wild B, Chiesa ST, Spranger J, Klostermann F, van den Akker EB, Trompet S, Mooijaart SP, Sattar N, Jukema JW, Lavrijssen B, Kavousi M, Ghanbari M, Ikram MA, Slagboom E, Kivimaki M, Langenberg C, Deanfield J, Eils R, Landmesser U (2022). Metabolomic profiles predict individual multidisease outcomes. Nature Medicine.

[CR4] Centers for Medicare & Medicaid Services Clinical Laboratory Improvement Amendments (CLIA) (Accessed November 2, 2023 2023). In https://www.cms.gov/medicare/quality/clinical-laboratory-improvement-amendments.

[CR22] U.S. Department of Health and Human Services Food and Drug Administration Center for Drug Evaluation and Research Center for Veterinary Medicine (2018). Bioanalytical Method Validation Guidance for Industry In https://www.fda.gov/files/drugs/published/Bioanalytical-Method-Validation-Guidance-for-Industry.pdf Accessed November 1, 2023 2023.

[CR5] European Medicines Agency (2022). ICH guideline M10 on bioanalytical method validation and study sample analysis. In https://www.ema.europa.eu/documents/scientific-guideline/ich-guideline-m10-bioanalytical-method-validation-step-5_en.pdf Accessed November 1, 2023 2023.

[CR6] Fuller H, Zhu Y, Nicholas J, Chatelaine HA, Drzymalla EM, Sarvestani AK, Julian-Serrano S, Tahir UA, Sinnott-Armstrong N, Raffield LM, Rahnavard A, Hua X, Shutta KH, Darst BF (2023). Metabolomic epidemiology offers insights into disease aetiology. Nat Metab.

[CR7] Gallo-Payet N, Battista MC (2014). Steroidogenesis-adrenal cell signal transduction. Compr Physiol.

[CR8] Han X, Lains I, Li J, Li J, Chen Y, Yu B, Qi Q, Boerwinkle E, Kaplan R, Thyagarajan B, Daviglus M, Joslin CE, Cai J, Guasch-Ferre M, Tobias DK, Rimm E, Ascherio A, Costenbader K, Karlson E, Mucci L, Eliassen AH, Zeleznik O, Miller J, Vavvas DG, Kim IK, Silva R, Miller J, Hu F, Willett W, Lasky-Su J, Kraft P, Richards JB, MacGregor S, Husain D, Liang L (2023). Integrating genetics and metabolomics from multi-ethnic and multi-fluid data reveals putative mechanisms for age-related macular degeneration. Cell Rep Med.

[CR9] Kachroo, P., Stewart, I. D., Kelly, R. S., Stav, M., Mendez, K., Dahlin, A., Soeteman, D. I., Chu, S. H., Huang, M., Cote, M., Knihtila, H. M., Lee-Sarwar, K., McGeachie, M., Wang, A., Wu, A. C., Virkud, Y., Zhang, P., Wareham, N. J., Karlson, E. W., Wheelock, C. E., Clish, C., Weiss, S. T., & Langenberg, C. J. A. Lasky-Su (2022). Metabolomic profiling reveals extensive adrenal suppression due to inhaled corticosteroid therapy in asthma. *Nature Medicine* 28, 814–822 10.1038/s41591-022-01714-5. 10.1038/s41591-022-01714-5 PMC9350737 35314841

[CR10] Klein, R., Myers, C. E., Buitendijk, G. H., Rochtchina, E., Gao, X., de Jong, P. T., Sivakumaran, T. A., Burlutsky, G., McKean-Cowdin, R., Hofman, A., Iyengar, S. K., Lee, K. E., Stricker, B. H., Vingerling, J. R., Mitchell, P., Klein, B. E., Klaver, C. C., & Wang, J. J. (2014). Lipids, lipid genes, and incident age-related macular degeneration: the three continent age-related macular degeneration consortium. Am J Ophthalmol 158, 513 – 24 e3 10.1016/j.ajo.2014.05.027. 10.1016/j.ajo.2014.05.027 PMC4138281 24879949

[CR11] Lains I, Kelly RS, Miller JB, Silva R, Vavvas DG, Kim IK, Murta JN, Lasky-Su J, Miller JW, Husain D (2018). Human plasma Metabolomics Study across all stages of age-related Macular Degeneration identifies potential lipid biomarkers. Ophthalmology.

[CR12] Lains, I., Zhu, S., Han, X., Chung, W., Yuan, Q., Kelly, R. S., Gil, J. Q., Katz, R., Nigalye, A., Kim, I. K., Miller, J. B., Carreira, I. M., Silva, R., Vavvas, D. G., Miller, J. W., Lasky-Su, J., Liang, L., & Husain, D. (2021). Genomic-metabolomic associations support the role of LIPC and glycerophospholipids in Age-Related Macular Degeneration. *Ophthalmol Sci*, *1*. 10.1016/j.xops.2021.100017. 10.1016/j.xops.2021.100017 PMC8353724 34382031

[CR13] Lasky-Su J, Kelly RS, Wheelock CE, Broadhurst D (2021). A strategy for advancing for population-based scientific discovery using the metabolome: The establishment of the Metabolomics Society Metabolomic Epidemiology Task Group. Metabolomics.

[CR14] Mozaffarian D, Katan MB, Ascherio A, Stampfer MJ, Willett WC (2006). Trans fatty acids and cardiovascular disease. New England Journal of Medicine.

[CR15] National Academy of Sciences, National Academy of Engineering, and Institute of Medicine (2005). *Facilitating Interdisciplinary Research*. Washington, DC: The National Academies Press. 10.17226/11153.

[CR16] North Carolina State University Research Development Office (2020). The Difference Between Multidisciplinary, Interdisciplinary, and Convergence Research. In https://research.ncsu.edu/rdo/the-difference-between-multidisciplinary-interdisciplinary-and-convergence-research/ Accessed November 2, 2023 2023.

[CR17] Oresic, M., McGlinchey, A., Wheelock, C. E., & Hyotylainen, T. (2020). Metabolic signatures of the exposome-quantifying the impact of exposure to Environmental Chemicals on Human Health. *Metabolites*, *10*. 10.3390/metabo10110454. 10.3390/metabo10110454 PMC7698239 33182712

[CR18] Page CP, Morley J (1999). Contrasting properties of albuterol stereoisomers. The Journal of Allergy and Clinical Immunology.

[CR19] Ridker PM (2003). Clinical application of C-reactive protein for cardiovascular disease detection and prevention. Circulation.

[CR20] Smith SW (2009). Chiral toxicology: It’s the same thing.only different. Toxicological Sciences.

[CR21] Sobrin L, Seddon JM (2014). Nature and nurture- genes and environment- predict onset and progression of macular degeneration. Progress in Retinal and Eye Research.

[CR23] Wieder C, Frainay C, Poupin N, Rodriguez-Mier P, Vinson F, Cooke J, Lai RP, Bundy JG, Jourdan F, Ebbels T (2021). Pathway analysis in metabolomics: Recommendations for the use of over-representation analysis. Plos Computational Biology.

[CR24] Wong WL, Su X, Li X, Cheung CM, Klein R, Cheng CY, Wong TY (2014). Global prevalence of age-related macular degeneration and disease burden projection for 2020 and 2040: A systematic review and meta-analysis. Lancet Glob Health.

[CR25] Zhang P, Carlsten C, Chaleckis R, Hanhineva K, Huang M, Isobe T, Koistinen VM, Meister I, Papazian S, Sdougkou K, Xie H, Martin JW, Rappaport SM, Tsugawa H, Walker DI, Woodruff TJ, Wright RO, Wheelock CE (2021). Defining the scope of Exposome studies and Research needs from a multidisciplinary perspective. Environmental Science & Technology Letters.

[CR26] Zhang Y, Tan W, Wang X, Zheng X, Huang Y, Li B, Meng Z, Gao Y, Qian Z, Liu F, Lu X, Shi Y, Shang J, Yan H, Zheng Y, Zhang W, Gu W, Qiao L, Deng G, Zhou Y, Hou Y, Zhang Q, Xiong S, Liu J, Duan L, Chen R, Chen J, Jiang X, Luo S, Chen Y, Jiang C, Zhao J, Ji L, Mei X, Li J, Li T, Zheng R, Zhou X, Ren H, Cheng X, Guo L, Li H (2023). Metabolic biomarkers significantly enhance the prediction of HBV-related ACLF occurrence and outcomes. Journal of Hepatology.

